# Differential expression patterns and clinical significance of estrogen receptor-α and β in papillary thyroid carcinoma

**DOI:** 10.1186/1471-2407-14-383

**Published:** 2014-05-29

**Authors:** Yanhong Huang, Wenwu Dong, Jing Li, Hao Zhang, Zhongyan Shan, Weiping Teng

**Affiliations:** 1 Department of Endocrinology and Metabolism, Institute of Endocrinology, The First Affiliated Hospital, China Medical University, Liaoning Provincial Key Laboratory of Endocrine Diseases, Shenyang 110001, P. R. China; 2 Department of General Surgery, The First Affiliated Hospital, China Medical University, Shenyang 110001, P.R. China

**Keywords:** ERα, ERβ1, Papillary thyroid cancer, Ki-67, Mutant P53, VEGF

## Abstract

**Background:**

The incidence of papillary thyroid cancer (PTC) is markedly higher in women than men during the reproductive years. *In vitro* studies have suggested that estrogen may play an important role in the development and progression of PTC through estrogen receptors (ERs). This study aimed to investigate the expression patterns of the two main ER subtypes, α and β1 (wild-type ERβ), in PTC tissue and their clinical significance.

**Methods:**

Immunohistochemical staining of thyroid tissue sections was performed to detect ER expression in female patients with PTC (n = 89) and nodular thyroid goiter (NTG; n = 30) using the Elivision™ plus two-step system. The relationships between ER subtype expression and clinicopathological/biological factors were further analyzed.

**Results:**

The positive percentage and expression levels of ERα were significantly higher in female PTC patients of reproductive age (18–45 years old; n = 50) than age-matched female NTG patients (n = 30), while ERβ1 exhibited the opposite pattern. There was no difference in ERα or ERβ1 expression between female PTC patients of reproductive age and those of advanced reproductive age (>45 years old; n = 39). In the female PTC patients of reproductive age, ERα expression level was positively correlated with that of Ki-67, while ERβ1 was negatively correlated with mutant P53. Furthermore, more patients with exclusively nuclear ERα expression had extrathyroidal extension (ETE) as compared with those with extranuclear ERα localization. VEGF expression was significantly decreased in female PTC patients of reproductive age with only nuclear ERβ1 expression when compared with those with extranuclear ERβ1 localization. In PTC patients of advanced reproductive age, neither ERα nor ERβ1 expression showed any correlation with that of Ki-67, mutant P53, VEGF, tumor size, TNM stage, ETE, or lymph node metastases.

**Conclusions:**

The differential expression patterns of the two ER subtypes between PTC and NTG indicate that ERα may be a useful immunohistochemical marker for differential diagnosis of PTC. The associations of ER subtype expression with Ki-67, mutant P53, VEGF expression and ETE in female PTC patients of reproductive age suggest that estrogen-activated ERα may mediate stimulatory effects on PTC growth and progression whereas ERβ1 has some inhibitory actions.

## Background

Papillary thyroid cancer (PTC) is the most common endocrine malignancy, and its incidence has rapidly increased in recent years [1]. PTC is usually indolent and curable after standard thyroidectomy in combination with TSH-suppressive levothyroxine therapy and radioiodine treatment, but can spread early to local lymph nodes. In addition, disease persistence and/or recurrence are common and associated with increased mortality [2]. According to the SEER 9 database of the National Cancer Institute in the USA, the female-to-male incidence ratio of PTC declined from more than five at ages 20–24 to 3.4 at ages 35–44, approaching one at ages 80+, and was greatest during reproductive age [3]. The predominance of PTC in females was observed in all geographical areas and ethnic groups [1,4]. Furthermore, Brindel et al. reported that the risk of thyroid cancer was significantly increased after natural or artificial menopause as compared with that at premenopausal status, and also with the number of births [5]. It has been found that estrogen can regulate the transcription of many cell proliferation-related genes [6-8]. There is also much evidence that estrogen has direct actions in thyroid cell lines originating from normal thyroid gland tissue and thyroid carcinoma by ER-dependent mechanisms, such as enhancement of proliferation, modulation of sodium-iodide symporter and thyroglobulin gene expression, and upregulation of matrix metalloproteinase 9 production [8-10]. The above findings indicate that the growth and progression of thyroid malignancies are influenced by female sex hormones, particularly estrogen [1]. Estrogen signaling is classically mediated upon the binding of two soluble intracellular receptors, ERα and ERβ [8]; the splice variant ERβ1 is wild-type ERβ. Several studies have investigated the expression of ER subtypes in thyroid cancers without consistent results as yet [8,11-13]. This may be owed to the existence of confounding factors in the subjects who were included in those analyses, such as age, gender and cancer types. Moreover, the precise contributions of the two ER subtypes have not been well understood [1,4]. This study was therefore performed to systemically investigate the expression patterns of ERα and ERβ1 in female PTC patients stratified by age, and to further analyze their relationships with important clinicopathological factors (e.g., tumor size) and biological markers (e.g., Ki-67).

## Methods

### Patients and tissue specimens

Thyroid specimens were obtained from 89 female Chinese patients with PTC, including 50 of reproductive age (18–45 years old), 39 of advanced reproductive age (>45 years old), and 30 of reproductive age with nodular thyroid goiter (NTG). All patients had a clinical duration of less than 3 years, and were admitted to our hospital for standard thyroidectomy from 2007 to 2010; those who underwent secondary surgery for PTC were excluded. Diagnoses were confirmed through histopathological examination by pathologists at our hospital. None of the PTC patients had a history of familial thyroid cancer or neck external irradiation. Patients with concomitant PTC and NTG as well as those only with papillary thyroid microcarcinoma were excluded from this study. All these criteria contributed to fewer confounding factors and greater homogeneity among the subjects. Clinicopathological data, such as tumor size, presence of extrathyroidal extension (ETE) and lymph node metastases (LNM), were retrieved from the relevant medical records. ETE was defined as invasion of adjacent organs or skeletal muscle outside the isthmus [14]. TNM stage was assessed according to the 7th edition of the tumor, node and metastasis system classification proposed by the American Joint Committee on cancer. This study was approved by the Institutional Review Board of the First Affiliated Hospital of China Medical University, and was in compliance with the Helsinki Declaration (AF-SOP-07-1.0-01). All patients gave written informed consent for participation in the study.

### Immunohistochemistry

Immunohistochemical (IHC) staining for ERα and ERβ1 was performed on 4-μm-thick formalin-fixed and paraffin-embedded sections of surgical specimens from PTC and NTG patients using the Elivision™ plus two-step system (Maxim Biotech Inc, Fuzhou, China), which has been proved superior to biotin-based SP and ABC detection systems due to large amounts of endogenous biotin present in thyroid [15,16]. Ki-67, mutant P53 and vascular epithelial growth factor (VEGF) are important growth-related biological markers for cancer. These proteins were examined in PTC specimens by IHC staining as described above. Briefly, the tissue sections were deparaffinized, rehydrated and subjected to microwave antigen retrieval in 10 mM citrate buffer (pH 6.0) for 20–25 min. Endogenous peroxidase was then blocked with 3% H_2_O_2_ for 10 min. After washing three times in phosphate buffered saline (PBS), the sections were incubated with a primary antibody against ERα (Clone 1D5, Dako; 1:200), ERβ1 (Clone PPG5/10, Serotec; 1:20), Ki-67 (Clone SP6, Abcam; 1:100), mutant P53 (Clone N235K N239Y, Bioss; 1:350) or VEGF (Clone EP1176Y, Abcam; 1:100) at 4°C overnight. Then, staining was performed according to the manufacturer’s instructions. Color reactions were performed with 3,3′-diamino-benzidine (DAB; Maxim Biotech Inc). The sections were then counterstained with hematoxylin, washed, dehydrated in alcohol, cleared in xylene, and mounted with coverslips. Appropriate positive and negative controls were used in each batch of staining experiments. Tissue sections from human breast cancer were used as positive controls for ERα and ERβ1. Negative control sections were incubated with normal mouse or rabbit IgG instead of a specific primary antibody.

### Review and scoring of stained tissue sections

Immunostained tissue sections were reviewed, scored and interpreted using the Allred score [17]. In brief, each section was carefully evaluated using light microscopy. A proportion staining score (PS) was assigned, which represented the estimated proportion of positive-staining cells as follows: 0, no staining; 1, <1⁄100; 2, 1⁄100 to 1⁄10; 3,1⁄10 to 1⁄3; 4, 1⁄3 to 2⁄3; and 5, >2⁄3. An intensity score (IS) was also assigned to represent the average intensity of positive cells in each section as follows: 0, no staining; 1, weak; 2, intermediate; and 3, strong. Finally, a total score (TS) was calculated from the sum of PS and IS (range 0, 2–8) to reflect the expression level. “Positive” staining was then defined as the TS ≥3. The sections were coded and analyzed separately by two independent investigators (Y.H. and W.D.), who were blinded to the other data regarding the sections. If discrepancies occurred, a third investigator (J.L.) evaluated the tissue sections to achieve a consistent result.

### Statistical analysis

Descriptive statistics were performed according to the distribution of variables. The Mann–Whitney U test was used for comparisons of quantitative variables between groups. The Chi-square test was used for comparisons of qualitative variables between groups. Relationships between ER subtype expression and biological markers were assessed using Spearman’s correlation analysis. All statistical analyses were performed with SPSS software (version 17.0, SPSS Inc., Chicago, IL, USA). Differences were considered statistically significant if *P <* 0.05.

## Results

### Expression patterns of ERα and ERβ1 in female PTC patients

**Figure 1:**
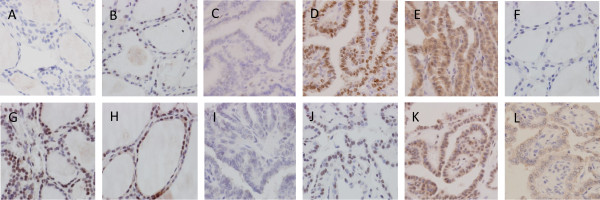
**Immunohistochemical staining of ERα and ERβ1 in PTC lesions and NTG tissue (400×).** ERα and ERβ1 expression was analyzed by immunohistochemical staining of formalin-fixed and paraffin-embedded thyroid tissue sections from patients with papillary thyroid cancer (PTC) and those with nodular thyroid goiter (NTG) using specific antibodies and the Elivision™ plus two-step system. **(A)** NTG tissue with negative ERα staining; **(B)** NTG tissue with positive nuclear ERα staining (total score 6); **(C)** PTC tissue with negative ERα staining; **(D)** PTC tissue with positive nuclear ERα staining (total score 8); **(E)** PTC tissue with positive nuclear and cytoplasmic ERα staining (total score 7); **(F)** NTG tissue with negative ERβ1 staining; **(G)** NTG tissue with positive nuclear ERβ1 staining (total score 8); **(H)** NTG tissue with positive nuclear and cytoplasmic ERβ1 staining (total score 7); **(I)** PTC tissue with negative ERβ1 staining; **(J)** PTC tissue with positive nuclear ER β1 staining (total score 6); **(K)** PTC tissue with positive nuclear and cytoplasmic ERβ1 staining (total score 7); **(L)** PTC tissue with positive cytoplasmic ERβ1 staining (total score 5).

Both the positive percentage and total score of ERα expression were significantly increased in female PTC patients of reproductive age as compared with that of age- and gender-matched NTG patients (Figure 1, Table 1). In addition, the subcellular localization of ERα was markedly different between PTC (26.7% with extranuclear expression) and NTG (no extranuclear expression). Notably, the expression level (i.e., total score) of ERβ1 was significantly lower in PTC lesions than NTG tissue (Figure 1, Table 1). Extranuclear ERβ1 expression was markedly more frequent in female PTC patients (85.4%) when compared with that of the NTG control group (17.9%) at reproductive age.

**Table 1:** **ERα/ERβ1 expression patterns between PTC and NTG tissues from female patients of reproductive age**^**a**^

			**PTC (n = 50)**	**NTG (n = 30)**	***P-*** **value**
**ERα**	**Positive percentage**		30/50 (60.0%)	2/30 (6.7%)	0.000
	**Total score**^**b**^		3.44 ± 2.80	0.27 ± 1.05	0.000
	**Subcellular localization**	Nu	22/30 (73.3%)	2/2 (100%)	0.000
		Nu + Cyto	6/30 (20.0%)	0	
		Cyto	2/30 (6.7%)	0	
**ERβ1**	**Positive percentage**		48/50 (96.0%)	28/30 (93.3%)	0.596
	**Total score**		6.36 ± 1.64	7.03 ± 1.85	0.001
	**Subcellular localization**	Nu	7/48 (14.6%)	23/28 (82.1%)	0.000
		Nu + Cyto	36/48 (75.0%)	5/28 (17.9%)	
		Cyto	5/48 (10.4%)	0	

^a^The female patients of reproductive age were 18–45 years old. ^b^Total score was calculated from the sum of the proportion staining score and the intensity score to reflect the expression level, which is given as mean ± standard deviation (S.D.). PTC, papillary thyroid cancer; NTG, nodular thyroid goiter; Nu, exclusively nuclear staining; Nu + Cyto, combined nuclear and cytoplasmic staining; Cyto, exclusively cytoplasmic staining.

The percentage of female PTC patients with extranuclear ERα expression was significantly lower at reproductive age (26.7%) than that at advanced reproductive age (61.1%). There was no significant difference in the positive percentage or expression level of either ER subtype between female PTC patients of reproductive age and those of advanced reproductive age (Table 2).

**Table 2:** **ERα/ERβ1 expression patterns in female PTC patients of reproductive age and of advanced reproductive age**^**a**^

			**Reproductive age (n = 50)**	**Advanced reproductive age (n = 39)**	***P*-value**
**ERα**	**Positive percentage**		30/50 (60.0%)	18/39 (46.2%)	0.194
	**Total score**^**b**^		3.44 ± 2.80	2.92 ± 3.35	0.342
	**Subcellular localization**	Nu	22/30 (73.3%)	7/18 (38.9%)	0.011
		Nu + Cyto	6/30 (20.0%)	3/18 (16.7%)	
		Cyto	2/30 (6.7%)	8/18 (44.4%)	
**ERβ1**	**Positive percentage**		48/50 (96.0%)	39/39 (100%)	0.206
	**Total score**		6.36 ± 1.64	6.69 ± 1.10	0.391
	**Subcellular localization**	Nu	7/48 (14.6%)	3/39 (7.7%)	0.404
		Nu + Cyto	36/48 (75.0%)	30/39 (76.9%)	
		Cyto	5/48 (10.4%)	6/39 (15.4%)	

^a^The female patients of reproductive age were 18–45 years old, while those of advanced reproductive age were all above 45 years old. ^b^Total score was calculated from the sum of proportion staining score and intensity score to reflect the expression level, which is given as mean ± standard deviation (S.D.). PTC, papillary thyroid cancer; Nu, exclusively nuclear staining; Nu + Cyto, combined nuclear and cytoplasmic staining; Cyto, exclusively cytoplasmic staining.

### Relationships between ERα/ERβ1 expression and clinicopathological/biological factors in female PTC patients

In female PTC patients of reproductive age or advanced reproductive age, there were no significant differences in the positive percentages or expression levels of the two ER subtypes when comparing tumor size (≤20 mm *vs >*20 mm), LNM (positive *vs* negative), ETE (positive *vs* negative) or TNM stage (I/II *vs* III/IV) (data not shown).

**Figure 2:**
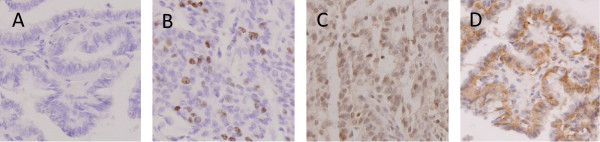
**Detection of Ki-67, mutant P53 and VEGF expression in PTC lesions by immunohistochemical staining (400×).** Formalin-fixed and paraffin-embedded PTC tissue sections were stained with the Elivision™ plus two-step system and specific antibodies against Ki-67, mutant P53 and VEGF. **(A)** PTC tissue stained with normal rabbit IgG instead of a specific primary antibody (negative control); **(B)** PTC tissue with positive Ki-67 staining (nuclear expression, total score 5); **(C)** PTC tissue with positive mutant P53 staining (combined nuclear and cytoplasmic expression, total score 7); **(D)** PTC tissue with positive VEGF staining (cytoplasmic expression, total score 7).

The expression of Ki-67, mutant P53 and VEGF in PTC lesions were examined as shown in Figure 2. Ki-67 is expressed in the nuclei of some PTC cells, and has been used as a marker for cell proliferation and prognosis in thyroid tumors, especially PTC [18]. Mutant P53 detection by IHC exhibited combined nuclear and cytoplasmic staining in PTC lesions, similar to the expression pattern reported in colorectal cancer and melanoma [19,20]. Mutant P53 has been recognized as a prognostic indicator for survival in PTC patients with a growth-promoting effect [21-23]. Similarly, cytoplasmic staining of VEGF was found in PTC tissues; VEGF is an important regulator of pathological neovascularization, and is especially involved in tumor growth and metastasis [24]. The total score of ERα expression was positively correlated with that of Ki-67 (r = 0.332, *P =* 0.028), while ERβ1 expression had a negative correlation with that of mutant P53 protein (r = −0.313, *P =* 0.039) in female PTC patients of reproductive age. The expression of neither ERα nor ERβ1 was significantly correlated with that of Ki-67, mutant P53 or VEGF in those of advanced reproductive age.

### Relationships between subcellular localization of ERα/ERβ1 and clinicopathological/biological factors in female PTC patients

As described above, ER subtypes were found to exhibit three types of expression patterns in PTC lesions—combined nuclear and cytoplasmic, exclusively nuclear or cytoplasmic localization. Among the female PTC patients of reproductive age, ETE occurred more frequently in those with exclusively nuclear ERα expression than with extranuclear localization of ERα (i.e., exclusively cytoplasmic or combined cytoplasmic and nuclear expression; Table 3). In addition, both the positive percentage and total score of VEGF expression were significantly decreased in samples with exclusively nuclear ERβ1 expression as compared with those demonstrating extranuclear localization of ERβ1 (Table 4). Among the female PTC patients of advanced reproductive age, no difference was found in the subcellular distribution of ERα or ERβ1 between tumors of different sizes, LNM, ETE or TNM stages (Table 3). No significant change was shown in the expression of Ki-67, mutant P53 or VEGF between the samples with exclusively nuclear ER subtype expression and those with its extranuclear localization (Table 4). The above findings suggest that estrogen may exert important effects on the growth and progression of PTC mainly through genomic actions mediated by ERα and ERβ1 localized to the nucleus.

**Table 3:** Associations between the subcellular localization of ERα/ERβ1 and clinicopathological factors in female PTC patients

**ERα**	**Tumor size**		**LNM**		**ETE**		**TNM**	
	**≤20 mm**	**>20 mm**	**+**	**-**	**+**	**-**	**I/II**	**III/IV**
**Reproductive age**^**a**^								
Nu (n = 28)	16/28 (57.1%)	12/28 (42.9%)	15/28 (53.6%)	13/28 (46.4%)	10/28 (35.7%)	18/28 (64.3%)	25/28 (89.3%)	3/28 (10.7%)
Nu + Cyto/Cyto (n = 8)	3/8 (37.5%)	5/8 (62.5%)	4/8 (50.0%)	4/8 (50.0%)	0	8/8 (100%)	8/8 (100%)	0
*P*-value	0.908		0.858		0.047		0.334	
**Advanced reproductive age**^**b**^								
Nu (n = 7)	5/7 (71.4%)	2/7 (28.6%)	2/7 (28.6%)	5/7 (71.4%)	4/7 (57.1%)	3/7 (42.9%)	4/7 (57.1%)	3/7 (42.9%)
Nu + Cyto/Cyto (n = 11)	4/11 (36.4%)	7/11 (63.6%)	5/11 (45.5%)	6/11 (54.5%)	2/11 (18.2%)	9/11 (81.8%)	6/11 (54.5%)	5/11 (45.5%)
*P*-value	0.109		0.474		0.087		0.914	
**ERβ1**	**Tumor size**		**LNM**		**ETE**		**TNM**	
	**≤20 mm**	**>20 mm**	**+**	**-**	**+**	**-**	**I/II**	**III/IV**
**Reproductive age**								
Nu (n = 7)	5/7 (71.4%)	2/7 (28.6%)	2/7 (28.6%)	5/7 (71.4%)	1/7 (14.3%)	6/7 (85.7%)	7/7 (100%)	0
Nu + Cyto/Cyto (n = 41)	19/41 (46.3%)	22/41 (53.7%)	25/41 (61.0%)	16/41 (39.0%)	15/41 (36.6%)	26/41 (63.4%)	38/41 (92.7%)	3/41 (7.3%)
*P*-value	0.374		0.110		0.247		0.460	
**Advanced reproductive age**								
Nu (n = 3)	2/3 (66.7%)	1/3 (33.3%)	0	3/3 (100%)	0	3/3 (100%)	3/3 (100%)	0
Nu + Cyto/Cyto(n = 36)	14/36 (38.9%)	22/36 (61.1%)	16/36 (44.4%)	20/36 (55.5%)	16/36 (44.4%)	20/36 (55.5%)	16/36 (44.4%)	20/36 (55.5%)
*P*-value	0.300		0.133		0.133		0.064	

^a^The female patients of reproductive age were 18–45 years old. ^b^Those of advanced reproductive age were all above 45 years old. PTC, papillary thyroid cancer; Nu, exclusively nuclear staining; Nu + Cyto, combined nuclear and cytoplasmic staining; Cyto, exclusively cytoplasmic staining; LNM, lymph node metastasis; ETE, extrathyroidal extension; TNM: tumor, node and metastasis staging system.

**Table 4:** Associations between subcellular localization of ERα/ERβ1 and biological markers in female PTC patients

**ERα**	**Ki-67**		**Mutant P53**		**VEGF**	
**Reproductive age**^**a**^						
**Subcellular localization**	**Positive percentage**	**Total score**^**c**^	**Positive percentage**	**Total score**	**Positive percentage**	**Total score**
Nu (n = 28)	27/28 (96.4%)	4.04 ± 1.07	21/28 (75.0%)	4.75 ± 2.98	26/28 (92.9%)	5.93 ± 2.14
Nu + Cyto/Cyto (n = 8)	8/8 (100%)	4.13 ± 0.64	5/8 (62.5%)	3.88 ± 3.36	8/8 (100%)	5.88 ± 1.81
*P*-value	0.588	0.983	0.486	0.562	0.437	0.683
**Advanced reproductive age**^**b**^						
Nu (n = 7)	6/7 (85.7%)	3.43 ± 1.51	4/7 (57.1%)	3.71 ± 3.50	3/7 (42.9%)	2.29 ± 3.09
Nu + Cyto/Cyto (n = 11)	8/11 (72.7%)	3.55 ± 2.30	8/11 (72.7%)	5.18 ± 3.37	8/11 (72.7%)	4.09 ± 3.05
*P*-value	0.518	0.121	0.494	0.182	0.205	0.205
**ERβ1**	**Ki-67**		**Mutant P53**		**VEGF**	
**Reproductive age**						
**Subcellular localization**	**Positive percentage**	**Total score**	**Positive percentage**	**Total score**	**Positive percentage**	**Total score**
Nu (n = 7)	6/7 (85.7%)	3.29 ± 1.60	6/7 (85.7%)	6.00 ± 2.77	4/7 (57.1%)	3.29 ± 3.15
Nu + Cyto/Cyto (n = 41)	40/41 (97.6%)	4.00 ± 1.00	28/41 (68.3%)	4.27 ± 3.11	41/41 (100%)	6.32 ± 1.47
*P*-value	0.147	0.227	0.349	0.118	0.000	0.015
**Advanced reproductive age**						
Nu (n = 3)	2/3 (66.7%)	2.67 ± 2.31	2/3 (66.7%)	4.67 ± 4.04	1/3 (33.3%)	2.33 ± 4.04
Nu + Cyto/Cyto (n = 36)	31/36 (86.1%)	3.81 ± 1.65	22/36 (61.1%)	4.14 ± 3.41	24/36 (66.7%)	3.61 ± 2.98
*P*-value	0.370	0.199	0.849	0.699	0.248	0.497

^a^The female patients of reproductive age were 18–45 years old. ^b^Those of advanced reproductive age were all above 45 years old. ^c^Total score was calculated from the sum of proportion staining score and intensity score to reflect the expression level, which is given as mean ± S.D. PTC, papillary thyroid cancer; Nu, exclusively nuclear staining; Nu + Cyto, combined nuclear and cytoplasmic staining; Cyto, exclusively cytoplasmic staining.

## Discussion

Both nuclear and extranuclear pools of ERα and ERβ have been identified [25]. The presence of ERs is fundamental for the direct action of estrogen in a given cell, which translocates from the cytoplasm into the nucleus after activation by the hormone. Membrane ERs possibly exist as a cytoplasmic pool tethered to the inner face of the plasma membrane bilayer through binding to proteins, such as caveolin-1 [25]. The importance of the subcellular localization of ERα/ERβ1 has been identified in a variety of cancers. Esophageal cancer invading through the esophageal wall was found to have a higher percentage of cells with cytoplasmic expression of ERβ1 than that only limited to the wall [26]. Furthermore, nuclear ERβ1 expression was associated with a favorable response to endocrine therapy in a cohort of 123 familial breast carcinomas [27]. ERα and ERβ distinctly regulate gene transcription among many cellular processes. ERα is well characterized as a mediator of cell proliferation, especially in breast cancer cells, driving cell proliferation in the presence of estrogen. ERβ includes five full-length subtypes (ERβ1-ERβ5) as a result of alternative splicing of the last coding exon. ERβ1 (the wild-type ERβ) has been found to exert opposing actions to ERα, and inhibits ERα-mediated proliferation in many cell types [28]. The expressions of ER subtypes and their clinical significance have been assessed in a wide range of different tumors, such as carcinomas of the breast and uterus [29,30]. PubMed searches revealed that approximately 16 studies of ER subtype expression in thyroid cancers including PTC have been reported since 1996. However, age, gender or tumor types were previously confounding factors in their statistical analyses, resulting in inconsistent findings [8]. There was also a lack of investigation of the subcellular localization of ER subtypes in these previous studies [8]. IHC assays with monoclonal antibodies are the most commonly used methods for establishing receptor status [8]. In this study, we systemically examined the expression patterns of ERα/β1 in PTC lesions and NTG tissues in female patients stratified by age. The cut-off point for age stratification in this study was selected to be 45 years, as estrogen levels decline with decreased ovulation in women above 45 years of age [31], who have approached or entered menopause [1,32]. Furthermore, 45 years of age is an important cut-off point for TNM staging of PTC [33]. Our study has shown that not only the expression level but also positive percentage of ERα in female PTC patients of reproductive age was significantly higher than that of age-matched female NTG patients. However, ERβ1 expression level in PTC was markedly decreased, although the positive percentage was similar between female PTC and NTG patients of reproductive age. In addition, extranuclear ERβ1 expression was significantly more frequent in PTC patients when compared with that of the NTG group. These findings indicate that ERα overexpression may stimulate the development of PTC whereas the constitutive expression of ERβ1 may play a suppressive role through its nuclear actions. Di Vito M et al. reported ERα overexpression in fine-needle aspiration biopsy-derived PTC specimens and cells using laser-capture microdissection followed by real-time quantitative PCR and western blotting [11]. Moreover, BCPAP cell line and cancer stem cells derived from PTC, which were analyzed under hypoxic conditions, showed a hypoxia-driven increase in ERα expression [11]. Inoue and colleagues also found that although PTC cells had low levels of ERα, following physiological estrogen stimulation the receptor level was significantly upregulated and cell proliferation was promoted [34]. Vannucchi and colleagues retrospectively followed 123 patients with differentiated thyroid cancer at different intervals during pregnancy. They found that patients with thyroid cancer detected during pregnancy were more likely to develop persistent and recurrent disease, and up to 87.5% of those patients had an ERα-positive tumor [35]. These findings also suggest that ERα may mediate the cancer-promoting effect of estrogen in PTC patients, and thus can be used as a marker of malignancy. The increase in the expression of ERα rather than ERβ in PTC cells induced by estrogen may be an important mechanism by which estrogen influences the development of the tumor [36]. Recently, ERβ has been reported to exhibit significantly higher expression in follicular thyroid adenoma than in follicular thyroid cancer (FTC), and to be a stronger differential diagnostic marker than Ki-67 [37]. Low ERβ expression appears to be correlated with poor survival in FTC [37]. In another study, compared with normal thyroid parenchyma, tumors gained ERα expression and lost that of ERβ [38]. Postsurgical serum thyroglobulin was higher in the ERα-positive tumors than the ERα-negative tumors, and ERβ-negative tumors showed more frequent vascular invasion than the ERβ-positive tumors [38]. Their study also suggests that ERβ may mediate inhibitory actions on the growth and progression of PTC, although its splice variants were not independently examined [38]. Our previous preliminary study showed that the expression patterns of ERβ1 and ERβ2 differed between PTC lesions and NTG tissue, and suggested that different ERβ splice variants may have differential roles in the pathogenesis of PTC [39]. Thus, the respective effects of the two ER subtypes and their splice variants on the development of PTC need to be separately investigated and analyzed to provide a basis for the use of corresponding ER agonists or antagonists in therapeutic and preventive approaches to PTC.

Although epidemiological and experimental studies have suggested a potential relationship between the development of thyroid malignancies and estrogens/ERs, their precise contributions in the initiation and progression of PTC have not been well understood [4]. In this study, we further analyzed the relationships of the two ER subtype expression patterns with some important clinicopathological factors and biological markers in female PTC patients of reproductive age and advanced reproductive age. Recently, some studies have focused on IHC markers and evaluated the expression of thyroid transcription factor-1, Ki-67, P63,P53 and VEGF in PTC lesions [40-42]. These proteins have been considered as useful markers reflecting the biological behavior and prognosis of PTC. In this study, we conducted IHC staining to analyze the associations between ERα/β1 expression patterns and that of Ki-67, mutant P53 and VEGF, and explored the roles of ER subtypes in the development of PTC. Ki-67 is a commonly used marker of proliferation in tumors and is universally expressed among proliferating cells and absent in quiescent cells. Previous studies have indicated that Ki-67 can predict disease-free survival and cause-specific survival of PTC patients as a prognostic marker [18,43], similar to the findings in breast cancer, an estrogen-related tumor [44]. Müssig et al. performed a retrospective analysis of 93 patients including 67 with PTC and 26 with FTC [45]. Ki-67 expression was significantly associated with tumor staging [45]. In this study, we found that ERα expression was positively correlated with that of Ki-67. Moreover, ETE occurred more frequently in those female PTC patients of reproductive age with exclusively nuclear ERα expression when compared with those exhibiting extranuclear localization of ERα. Our findings suggest that increased nuclear ERα expression may stimulate the growth of PTC and is associated with adverse clinical outcome. Several *in vitro* studies have demonstrated estradiol-induced proliferation of thyroid cells using the most commonly used assays, such as bromodeoxyuridine (BrdU) incorporation, 3-(4,5-dimethylthiazol-2-yl)-2,5-diphenyl tetrazolium bromide (MTT), ^3^H-thymidine incorporation, and trypan blue solution [8]. Furthermore, the actions of ER-subtype specific agonists on thyroid cancer cell lines have been studied *in vitro*: propyl-pyrazole-triol (PPT, ERα*-*specific) stimulated cell proliferation, while diarylpropionitrile (DPN, ERβ*-*specific) had an inhibitory effect [46]. It has been proposed that ERα and ERβ may play different roles in the development of thyroid carcinoma: ERα activation promotes cell proliferation and growth, while ERβ activation induces apoptosis and mediates other suppressive actions of estrogen [8,47]. Wild-type P53 protein is an important tumor suppressor that can regulate many cellular activities including cell cycle arrest, apoptosis, and angiogenesis. It is not only involved in the control of tumorigenesis, but also extends to other stages of cancer development, such as tumor invasion and metastasis [21]. Mutant P53 has been noted in a variety of human malignancies, which loses its suppressive activity and gains specific ‘mutant functions’ [48]. The dominant oncogenic properties of mutant P53 have been recognized through its growth-promoting effects associated with tumor progression. Wild-type P53 has a short intracellular half-life and is usually undetectable by IHC, whereas mutant P53 proteins have a longer half-life, which results in a sufficient increase in the amount detectable by IHC. Balta et al. reported that mutant P53 expression in PTC lesion was significantly increased as compared with that of benign thyroid tissue [22]; and its positive percentage in PTC varied from 41.2 to 76.0% [40,49]. Mutant P53 has been identified as a prognostic indicator for survival in PTC [22,23,50]. Significant correlations were reported between P53 protein expression detected by immunohistochemistry in the primary tumor of PTC and tumor size, the presence of lymph node metastasis and the mean number of lymph node metastases [50]. In addition, it has been suggested that *P53* gene mutations trigger progression from differentiated to anaplastic carcinoma in human thyroid glands [51]; P53 is particularly hypermutable in thyroid cancer [52]. Classical P53 function depends on its nuclear localization, and gene mutation is one of the important mechanisms by which P53 is sequestered to the cytoplasm [19,53]. Although most investigators consider nuclear expression to be an indication of *P53* gene mutation, cytoplasmic accumulation of mutant P53 is actually present in some tumors, including colorectal cancer, lung tumors and melanoma [19,20]. In colorectal cancer, tumors with P53 accumulation in both the nucleus and cytoplasm tend to have a higher mutation rate and more multiple mutations, accompanied by the most unfavorable outcome [19]. Ardito G et al. reported that P53 protein detectable by IHC showed a prevailing cytoplasmic localization including exclusive cytoplasmic and nuclear/cytoplasmic positivity in PTC lesions [54]. In our study, by IHC staining with mutant P53 specific primary antibody we found that it was localized to both the nucleus and cytoplasm of PTC cells. Furthermore, the expression level of ERβ1 was negatively correlated with that of mutant P53 in female PTC patients of reproductive age, indicating that decreased ERβ1 may be associated with PTC progression. The interactions between mutant P53 and ER have been suggested to play a potential role in mammary tissue homeostasis and cancer formation. Using human colon carcinoma HCT116 cells, Menendez et al. found that ERβ bound to a ligand and acting *in cis* is required or can stimulate the function of selected P53 mutants toward at least some half and full site response elements and also an endogenous gene target [55]. VEGF is known as an important regulator of pathological neovascularization and is especially involved in tumor growth and metastasis. High VEGF expression is associated with tumor aggressiveness (i.e., metastatic involvement and recurrence) and poor survival of patients with thyroid carcinomas [24,56]. Tian et al. reported that the frequency of VEGF expression was higher in PTC tissue as compared with that of adjacent normal follicular epithelium [57]. Also, its expression was significantly more frequent in PTC with LNM than without LNM [57]. In this study, we found that both the positive percentage and total score of VEGF expression were significantly decreased in female PTC patients of reproductive age with exclusively nuclear ERβ1 expression as compared with those with extranuclear localization of ERβ1, suggesting that estrogen may suppress VEGF expression through genomic actions mediated by ERβ1 localized to the nuclei of PTC cells. It has been reported that a follicular thyroid cancer cell line, ML-1, secreted more VEGF after estrogen stimulation, likely as a result of ER signaling [9]. In an animal model of ischemic stroke, ERβ contributed to the reduction of vasogenic edema via the inhibition of VEGF production [58]. ERβ in the nucleus has been found to attenuate the hypoxic induction of VEGF mRNA by directly decreasing HIF-1α binding to the VEGF gene promoter [59]. The reports described above indicated the potential effects of ERβ1 activation on mutant P53 and VEGF, consistent with our findings. Our study further suggests that ERβ1 may mediate some suppressive actions on the growth, invasion and metastasis of PTC, as found in esophageal and breast cancers [26,60].

## Conclusions

Our current study has demonstrated the differential expression patterns of ERα and ERβ1 in PTC and NTG. These findings indicate that the detection of ER subtypes may help in the differential diagnosis for PTC, and ERα especially, may be a useful IHC marker. The associations of ERα/ERβ1 expression with that of Ki-67, mutant P53, VEGF and ETE found in female PTC patients of reproductive age suggest that estrogen-activated ERα may mediate stimulatory effects on the growth and progression of PTC, whereas ERβ1 exerts some inhibitory actions. The increased ERα and decreased ERβ1 expression levels may play important roles in the pathogenesis of PTC among female patients of reproductive age. These findings provide further evidence to support the development of alternative therapeutic and preventive approaches to PTC with ERα-specific antagonists or ERβ1-specific agonists. However, we recognize that our cohort was relatively small and the follow-up period was not very long, and thus may not fully reveal the association between ERα/ERβ1 expression and postoperative recurrence in PTC patients. Long-term prospective observations of PTC patients under both standard thyroidectomy and standard postoperative management are required, and will be initiated in the near future. The results will help to better illustrate the relationship between ER subtype expression and the prognosis of PTC patients.

## Abbreviations

ER: Estrogen receptor; PTC: Papillary thyroid cancer; NTG: Nodular thyroid goiter; ETE: Extrathyroidal extension; LNM: Lymph node metastases; IHC: Immunohistochemical; VEGF: Vascular epithelial growth factor; PBS: Phosphate buffered saline; DAB: 3,3′-diamino-benzidine; PS: Proportion staining score; IS: Intensity score; TS: Total score; BrdU: Bromodeoxyuridine; MTT: 3-(4,5-dimethylthiazol-2-yl)- 2,5-diphenyl tetrazolium bromide; PPT: Propyl-pyrazole-triol; DPN: Diarylpropionitrile; Nu: Exclusively nuclear staining; Nu + Cyto: Combined nuclear and cytoplasmic staining; Cyto: Exclusively cytoplasmic staining; TNM: tumor, node and metastasis staging system.

## Competing interests

The authors declare that they have no competing interests.

## Authors’ contributions

LJ had the idea for this research and took responsibility for the design of this work. HYH and DWW made substantial contributions in acquisition of data, laboratory analyses and data interpretation and performed all statistical analyses. LJ, HYH and DWW wrote the manuscript. ZH, SZY and TWP were involved in revising the manuscript and have given final approval of the version to be published. All authors read and approved the final manuscript.

## Pre-publication history

The pre-publication history for this paper can be accessed here:

http://www.biomedcentral.com/1471-2407/14/383/prepub
